# Monitoring the Impact of Air Quality on the COVID-19 Fatalities in Delhi, India: Using Machine Learning Techniques

**DOI:** 10.1017/dmp.2020.372

**Published:** 2020-10-12

**Authors:** Jasleen Kaur Sethi, Mamta Mittal

**Affiliations:** University School of Information Communication and Technology (USICT), Guru Gobind Singh Indraprastha University (GGSIPU), New Delhi, India; Department of Computer Science and Engineering, G. B. Pant Government Engineering College, Okhla, New Delhi, India

**Keywords:** air pollutants, COVID-19, Decision Trees, Linear Regression, machine learning, Random Forest

## Abstract

**Objective:**

The focus of this study is to monitor the effect of lockdown on the various air pollutants due to the coronavirus disease (COVID-19) pandemic and identify the ones that affect COVID-19 fatalities so that measures to control the pollution could be enforced.

**Methods:**

Various machine learning techniques: Decision Trees, Linear Regression, and Random Forest have been applied to correlate air pollutants and COVID-19 fatalities in Delhi. Furthermore, a comparison between the concentration of various air pollutants and the air quality index during the lockdown period and last two years, 2018 and 2019, has been presented.

**Results:**

From the experimental work, it has been observed that the pollutants ozone and toluene have increased during the lockdown period. It has also been deduced that the pollutants that may impact the mortalities due to COVID-19 are ozone, NH_3_, NO_2_, and PM_10._

**Conclusions:**

The novel coronavirus has led to environmental restoration due to lockdown. However, there is a need to impose measures to control ozone pollution, as there has been a significant increase in its concentration and it also impacts the COVID-19 mortality rate.

An epidemic that occurs over a very large area worldwide and affects a large population is called a *pandemic*. An influenza pandemic is characterized by a widespread transmission occurring worldwide.^1^ Some of the influenza epidemics that have been recorded are the severe acute respiratory syndrome coronavirus (SARS-CoV)^2^ in 2002 and the Middle East respiratory syndrome coronavirus (MERS-CoV) that emerged in Saudi Arabia in 2012. Another such swine origin influenza pandemic known as A (H1N1) emerged in March 2009 that led to about 3200 deaths worldwide by September 2009, for which later a vaccine was developed.^3^

An outbreak of pneumonia of unknown origin occurred in Wuhan, China. This outbreak was later attributed to a novel coronavirus, SARS-CoV-2, responsible for causing the 2019 coronavirus disease (COVID-19).^4,5^ The virus affects the lower respiratory tract and leads to serious illness in older people or in those with diabetes, heart, or respiratory problems.^6^ Initially, all the cases in other countries were attributed to infection from China but later extended to a number of countries like Japan, South Korea, Italy, and the United States. COVID-19 has affected countries in all continents and was thus declared a pandemic by the World Health Organization (WHO) on March 11, 2020.^7^ As per the data by Johns Hopkins University, as of April 10, 2020, more than 1.49 million infections and 90 000 deaths were reported due to COVID-19 worldwide.^8^

The second most populated country in the world is India, and thus any pandemic in India which is unrestrained can affect about one-sixth of the population in the world.^9^ As per the Ministry of Health and Family Welfare (MoHFW), Government of India, by April 10, 2020, there have been 6039 active cases and 206 deaths due to COVID-19 in India. The highest number of cases has emerged in Maharashtra with 1364 cases and 97 deaths, followed by Delhi with 898 cases and 13 deaths.^10^ To mitigate the spread of COVID-19, MoHFW in January 2020 advised to abstain the travel to China, and thermal screening of all passengers returning from various countries was carried out at 21 airports across India. Due to the absence of any vaccine or medicine for COVID-19, to slow down the spread of the virus, there is a need for early detection and self-isolation of infected patients along with quarantine and hand hygiene, as the transmission occurs through droplets from coughing and sneezing.^11^ The WHO^12^ and MoHFW have issued the following guidelines to mitigate the spread of COVID-19:

- To wash hands frequently using soap and water or alcohol-based hand rub
- To cover the mouth and nose with disposable tissue or handkerchief while sneezing or coughing and throw the used tissues immediately
- To avoid public gatherings and practice social distancing
- To visit a doctor while wearing a mask if one experiences fever, cough, and difficulty breathing^13^

Apart from the above recommendations, the Indian Government took several measures to contain the spread of COVID-19. The state governments shut all schools, colleges, cinema halls, and malls. The Directorate General of Civil Aviation canceled all international flights landing in India starting March 22, 2020, and all domestic flights starting March 25, 2020. A 14-hour public curfew on March 22 followed by a national lockdown of 21 days was ordered by the Indian Prime Minister starting March 25, 2020.

As per the WHO global pollution database of the year 2016, based on the particulate matter concentration, 14 out of the 15 most polluted cities in the world are in India.^14^ COVID-19 affects the lower respiratory tract, so air pollution could further impact the deaths due to the virus. Thus, there is a need to enforce regulations to control the air pollution both during and after a lockdown. The influence of a lockdown due to the COVID-19 pandemic on the air quality in India has been studied by many researchers. Mahato et al.^15^ studied the air quality of Delhi amidst the lockdown due to COVID-19. Seven pollutant concentrations for 34 stations in the city were studied, and it was found that PM_2.5_, PM_10_, CO, and NO_2_ have maximum reduction in comparison to the pre-lockdown phase. Kambalagere^16^ analyzed the air quality index (AQI) of Bengaluru before and after the lockdown and found that the air quality improved from a hazardous level during the lockdown. Contini and Costabile^17^ studied the influence of atmospheric pollutants on COVID-19 and its mortality rate. It was found that PM_2.5_ and PM_10_ concentrations may increase the vulnerability to COVID-19. Therefore, air pollution along with other factors like population, age, density, and meteorological parameters should be considered in the future to determine the importance of these factors in the mortality rate due to COVID-19. Mitra et al.^18^ compared the concentration of CO_2_ at 12 monitoring stations in Kolkata during April, 2019, and during the lockdown on April 2020. Temporal variation of CO_2_ was observed, but no statistically significant variation existed between various monitoring stations. Furthermore, some sites did not lead to a decrease in CO_2_ as its previous concentration was low due to widespread floral species. Sharma et al.^19^ studied the restricted emissions in the lockdown due to COVID-19 pandemic and analyzed the concentration of 6 criteria pollutants from mid-March to mid-April 2017 to 2020 in over 22 cities of India. While all the pollutants and the AQI decreased significantly, an increase in ozone was observed. Furthermore, the WRF-AERMOD model was applied for predicting PM_2.5_ concentration of Delhi with actual meteorological parameters and the events of November 2019, and it was found that there was an increase of 33% in PM_2.5_ concentration. Though an overall decrease in the concentration of the majority of pollutants has been observed during the lockdown period, the influence of these pollutants on COVID-19 and its mortality rate needs to be explored further. In this study, the impact of various pollutants in Delhi on fatalities due to COVID-19 has been studied, and a further comparison between the air pollution levels over the past 2 years has been performed.

## METHODS

The comparison of air pollutants in Delhi during the lockdown period in the previous 2 years and the methodology used to assess the impact of air pollutants on COVID-19 fatalities has been shown in the following section.

### Air Quality of Delhi

**FIGURE 1:**
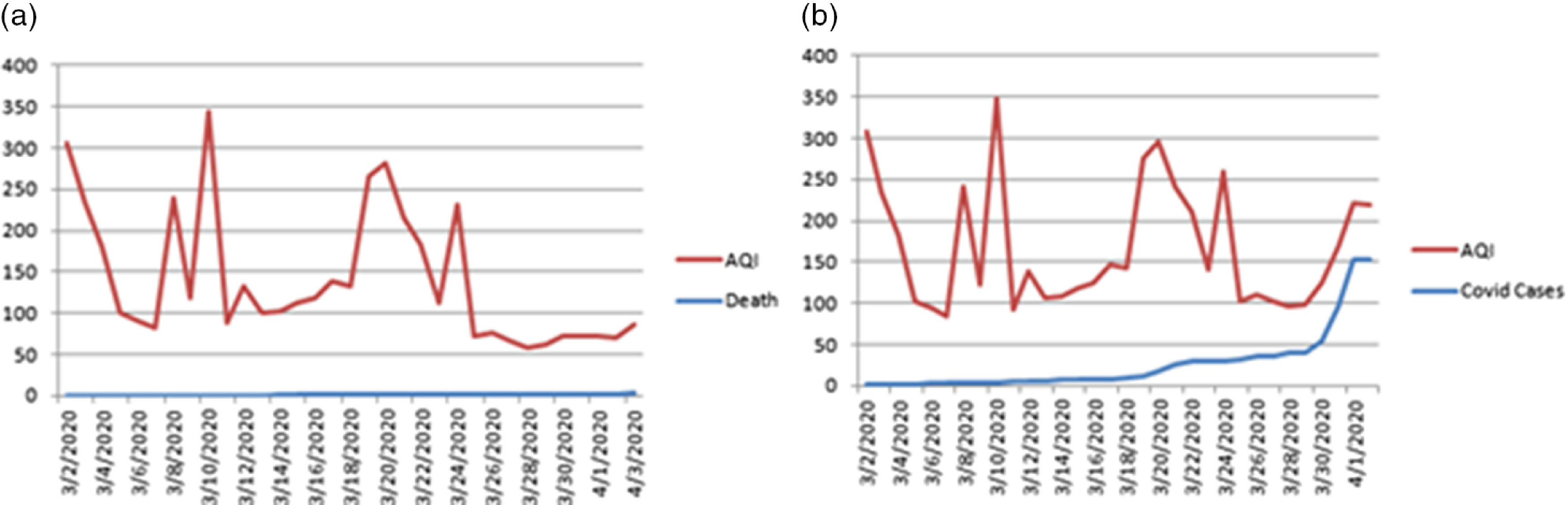

Air quality is assessed by the AQI tool that maps the concentration of a number of pollutants (CO, SO_2_, PM_2.5_, Ozone, and NO_2_, etc) into a single value. To compute this index, a subindex of each pollutant is first calculated and then these subindices are combined in weighted additive form. AQI has been divided into 6 categories in India: Good (0–50), Satisfactory (51–100), Moderately Polluted (101–200), Poor (201–300), Very Poor (301–400), and Severe (401–500).^20,21^ Air pollution is the cause of respiratory and other impacts on human health and increases the mortality and morbidity rates.^22,23^ The National Capital Territory (NCT) of Delhi is one of the most polluted cities of the world. Major sources of poor air quality in Delhi include industrial activity and the emission from vehicles which are increasing at a rate of 7% every year as per the Transport Department, NCT, of Delhi.^24^ The first positive case of COVID-19 was reported in Delhi on March 2, 2020. Following that, all the primary schools were shut from March 6, 2020, and all colleges, schools, and cinema halls since March 13, 2020. Subsequently, all the domestic and international flights were canceled, and a 21-day lockdown was announced by the Indian Prime Minister to mitigate the spread of COVID-19, which led to the closure of all factories and vehicles, as most of the people were restricted to their homes. Thus, the air quality of Delhi has improved drastically. The line graphs between the AQI and the number of deaths and between AQI and the number of COVID-19 cases in Delhi are shown in Figure 1.

**FIGURE 2:**
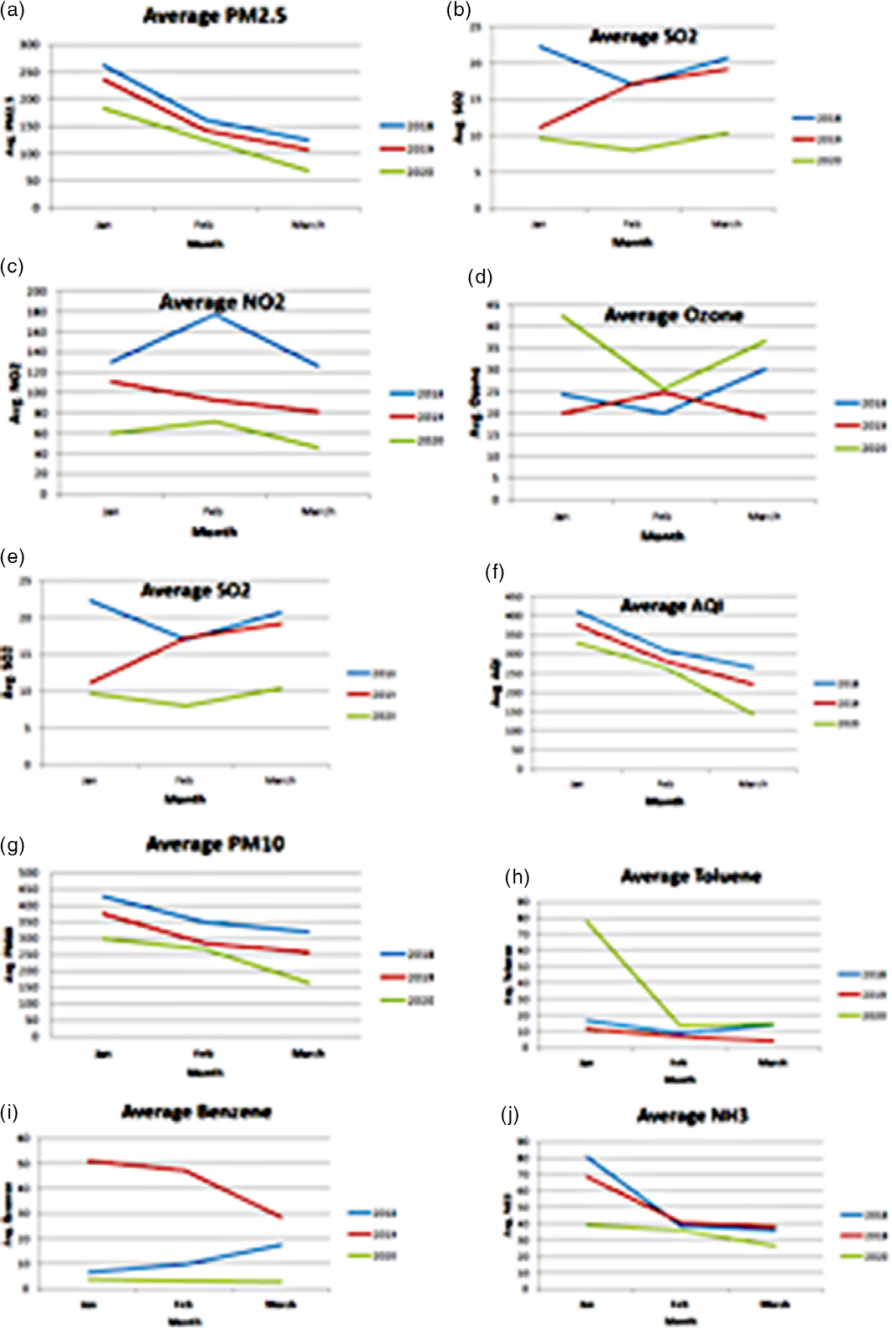

From Figure 1, it has been observed that the air quality of Delhi improved significantly as the number of COVID-19 cases increased. It has also been noted that gradual improvement occurred through March 2020, especially after the lockdown was declared after March 21, 2020. Furthermore, the monthly averages of AQI and the pollutants, namely, PM_2.5_, CO, NO_2_, SO_2_, ozone, PM_10_, toluene, benzene, and NH_3_, in January, February, and March 2020, have been plotted and compared with the averages of the previous 2 years, as shown in Figure 2.

From the plots in Figure 2, it has been observed that the overall air quality for the year 2020 showed improvement over the previous 2 years starting in January. The reason for this can be attributed to COVID-19, as its first positive case was reported in India in January 2020. Furthermore, it has also been noted that the concentration of the pollutants has decreased significantly, apart from ozone and toluene. The average concentration of all the pollutants and AQI in 2020 compared with that of the previous 2 years for the months of January, February, and March has been summarized in Table 1.

**TABLE 1:** Average Concentration of Various Pollutants and AQI in Delhi

	Jan			Feb			March		
Average Pollutant Concentration	2018	2019	2020	2018	2019	2020	2018	2019	2020
**PM**_**2.5**_	261.64	235.87	182.49	161.27	142.47	124.17	125.57	106.83	67.92
**CO**	3.10	3.17	2.51	2.78	1.89	2.11	1.83	2.04	1.85
**NO**_**2**_	130.62	110.84	60.45	177.34	93.07	71.46	126.77	81.56	46.04
**Ozone**	**24.22**	**20.04**	**42.54**	**20.09**	**24.74**	**25.70**	**30.25**	**18.85**	**36.65**
**SO**_**2**_	22.24	11.17	9.66	17.02	17.20	7.96	20.76	19.16	10.34
**AQI**	412.27	377.75	330.19	310.13	281.54	264.11	265.45	222.05	144.13
**PM**_**10**_	429.40	375.04	299.78	348.30	284.51	266.33	319.65	258.51	165.11
**Toluene**	**17.05**	**11.37**	**78.04**	**8.73**	**6.85**	**13.43**	**13.93**	**4.14**	**14.85**
**Benzene**	6.74	50.92	3.81	9.86	47.34	3.24	17.72	28.34	2.74
**NH**_**3**_	80.83	68.66	38.90	38.69	40.74	35.84	35.90	38.06	26.14

From Table 1, it has been noted that the highest percentages of decrease, 63.68%, 56.56%, and 93.16%, exist for the concentrations of NO_2_, SO_2,_ and benzene, respectively. This occurs as the main sources of these pollutants are vehicular emissions and industries, which are shut due to lockdown. Furthermore, it can be inferred that, in March 2020, there has been a percentage increase of 21.14% since 2018 and 94.42% since 2019 in the ozone concentration. This occurs as ozone is a secondary pollutant that is formed from NO_2_, which is present due to the limited vehicles on roads during lockdown. An increase of 95.98% exists for toluene since February 2019.

### Description of the Machine Learning Techniques

In this paper, feature selection has been carried out using various machine learning techniques, namely, Decision Trees, Linear Regression, and Random Forest. These techniques have been explained in detail in the following texts.

#### Decision Trees

Decision Trees represent the parameters by a node in the tree, and the values of these parameters are represented by the respective branches of the node and perform division of input based on the values of the various parameters.^25^ The design of a decision tree classifier depends on the design of the tree structure, feature subsets at each internal node, and the decision rule to be used at each node. Error rates, number of nodes in the tree, and information gain are some of the criteria for the design of the tree structure. Branch and bound technique and greedy algorithm are used for feature subset selection, whereas entropy and information-based approaches are generally used as a decision rule at each node.^26^

#### Linear Regression

Linear Regression represents a dependent variable based on the linear combination of various independent variables.^27^ A scatter plot is constructed and a correlation is computed between the response variable and the predictors. The regression coefficient that is the intercept and slope coefficient are finally calculated to find the regression line, which determines the predicted value of the response variable.^28^

#### Random Forest

It is based on the forecast of a number of trees where each tree is trained independently and then averaging the values of the result.^29^ To construct Random Forest, samples equal to the number of trees are drawn from the dataset, and a tree is grown by choosing the best split amongst all the input variables at each node. Prediction is carried out by aggregation of predictions of all the sample trees. For regression is an average prediction and for classification the majority voting amongst predictions is considered.^30^

### Methodology to Monitor Impact of Air Quality on COVID-19 Fatalities

**FIGURE 3:**
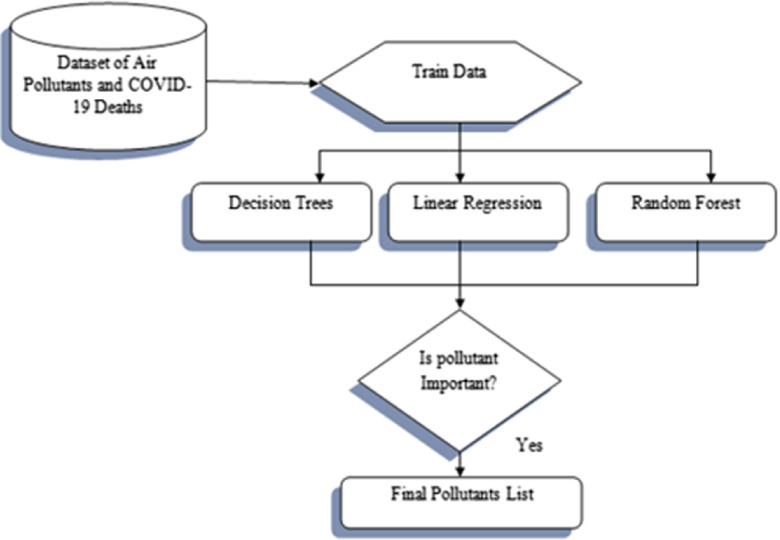

Recent studies have suggested that air pollution could lead to an increase in COVID-19 deaths as the virus tends to weaken the respiratory system. Thus, there is a need to identify the pollutants in Delhi that affect the novel coronavirus so that measures to control pollution could be enforced. In this work, feature selection techniques based on machine learning are employed to find the various pollutants that influence the COVID-19 deaths. The methodology to monitor the impact of air quality on deaths due to COVID-19 has been summarized in Figure 3.

- The dataset with AQI and various pollutants (PM_2.5_, CO, NO_2_, SO_2_, ozone, PM_10_, toluene, benzene, and NH_3_) as predictors and COVID-19 deaths in Delhi as response are collected from the Central Pollution Control Board^31^ and MoHFW website.
- To pre-process the dataset, the missing values of various pollutants have been replaced by their mean. The dataset has then been split into 70% train and 30% test set.
- Feature selection has been carried out using Decision Trees, Linear Regression, and Random Forest. These techniques are used to build a model on the train data and compute the feature importance on the test data.
- To train the data using the machine learning techniques, first the tuning parameters for each model are chosen and then resampling is performed using the cross-validation method. The tuning parameters used for Decision Trees, Linear Regression, and Random Forest are the complexity parameters used to select the optimal tree size, the intercept of the regression line, and the number of input parameters used for splitting at each node, respectively.
- The importance is computed on the test data based on the model statistic. A feature is of importance if a reduction in the model statistic is noticed when that feature is added to the model. For Linear Regression, the t-statistic is used, and for Decision Tree and Random Forest, the mean square error is used as the model statistic.
- The final pollutants are then selected by the machine learning techniques that influence COVID-19 deaths, and various measures to control pollution could be enforced to handle the COVID-19 crisis.

## RESULTS

Amid the lockdown, the concentration of most of the pollutants has reduced. To monitor the influence of the pollutants on COVID-19 and its mortality rate, various machine learning techniques have been applied to extract the features. A correlation between the pollutants and the deaths due to COVID-19 in Delhi has been depicted in Table 2.

**TABLE 2:** Correlation Between Pollutants and COVID-19 Deaths

Pollutant	Correlation Between Pollutant and COVID-19 Deaths
PM2.5	-0.28468
CO	-0.04037
NO2	-0.45827
Ozone	0.520042
SO2	0.406352
PM10	-0.33557
Toluene	-0.02686
Benzene	-0.08389
NH3	0.450899
AQI	-0.34013

From Table 2, it can be inferred that COVID-19 deaths have a positive correlation with ozone, SO_2_, and NH_3_. Further, 3 machine learning techniques – Decision Trees, Linear Regression, and Random Forest – have been applied to extract the pollutants that influence the death due to the novel coronavirus. The importance of each pollutant, as computed by various techniques, has been displayed in Table 3.

**TABLE 3:** Relative Importance of Pollutants Based on Machine Learning Techniques

Decision Trees		Linear Regression		Random Forest	
**Pollutant**	**Importance**	**Pollutant**	**Importance**	**Pollutant**	**Importance**
**NO**_**2**_	**100**	**NH**_**3**_	**100**	**Ozone**	**100**
**Ozone**	**94.94**	CO	37.07	**NH**_**3**_	**93.36**
**NH**_**3**_	**81.9**	**Ozone**	**36.33**	**NO2**	**63.65**
**PM**_**10**_	**68.97**	**PM**_**10**_	**30.14**	**PM**_**10**_	**48.94**
PM_2.5_	52.53	**NO2**	**27.99**	SO_2_	40.63
SO_2_	26.63	PM_2.5_	22.12	AQI	32.58
CO	0	AQI	21.13	PM_2.5_	23.92
Toluene	0	SO_2_	12.3	CO	14.28
AQI	0	Toluene	11.41	Toluene	14.04
Benzene	0	Benzene	0	Benzene	0

**FIGURE 4:**
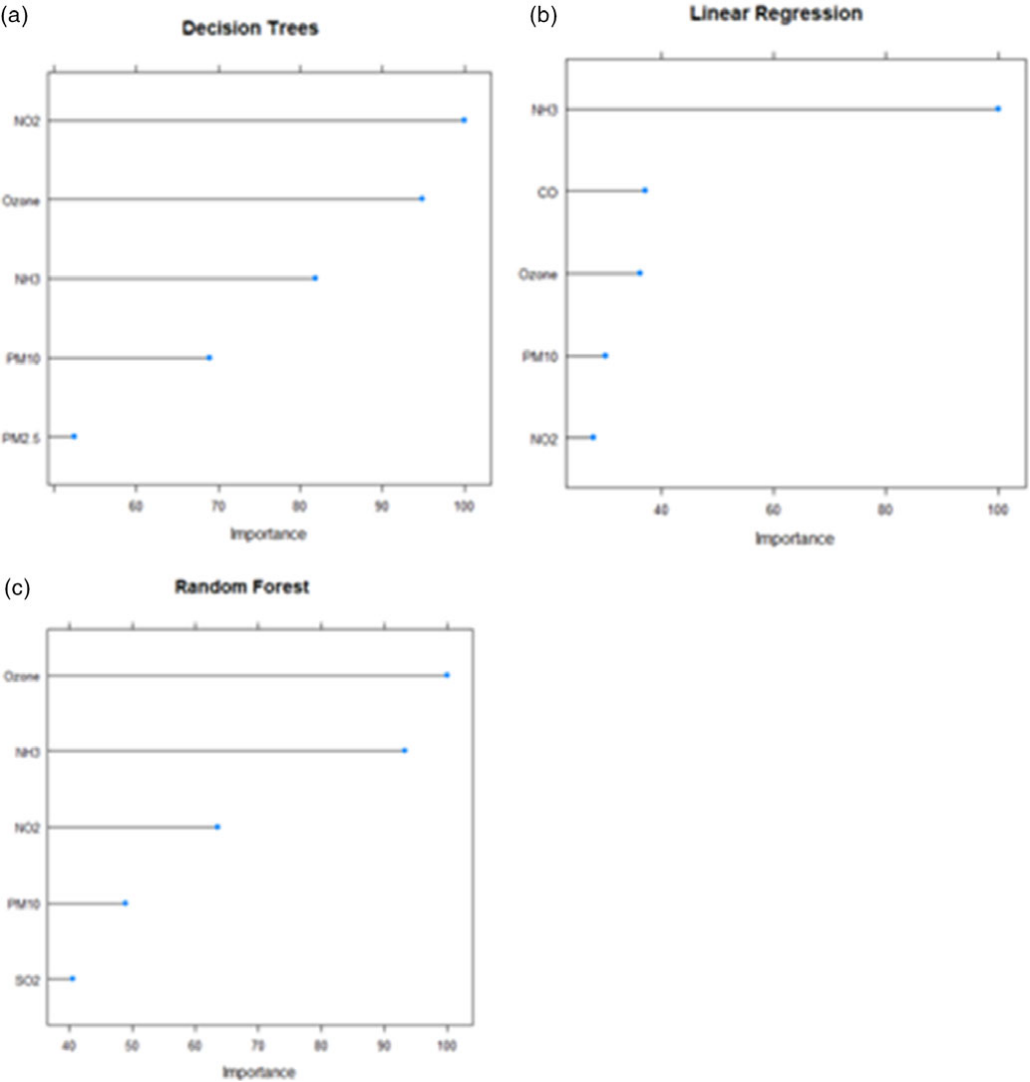

Table 3 depicts that the most important pollutant by Decision Trees, Linear Regression, and Random Forest is NO_2,_ NH_3_, and ozone, respectively. The 5 most important indicators as computed by different machine learning techniques have been shown in Figure 4.

From Figure 4, it can be inferred that the important pollutants that have been found by all 3 techniques are ozone, NH_3_, NO_2_, and PM_10._ Thus, there is a need to take measures to control these pollutants during and after lockdown, as they influence COVID-19 deaths.

## DISCUSSION

In December 2019, an outbreak of pneumonia of unknown origin occurred in Wuhan, China. This outbreak was later identified to be caused by a novel coronavirus responsible for COVID-19. The outbreak has affected countries in all continents and thus has been declared a pandemic by the WHO.^32^ The virus leads to serious illness in older people or ones with diabetes, heart, or respiratory problems and would lead to more COVID-19 deaths in areas with poor air quality. Thus, there is a need to enforce regulations to curb pollutions during and after lockdown. In this work, it has been found that ozone and toluene have increased in the lockdown period, and the pollutants that may impact the COVID-19 fatalities are ozone, NH_3_, NO_2_, and PM_10._ Ozone is of significance, as it is one of the pollutants that has increased in the lockdown period and considerably may affect the mortality due to the novel coronavirus; also, it has been noted that high levels of ozone concentration lead to mental conditions such as depression.^33^ Thus, there is a need to impose measures to control the ozone pollution. According to the Centre for Science and Environment, the areas with lowest concentrations of NO_2_ have high ozone levels as nitrogen oxides, volatile organic compounds (VOC), and other gases react in the presence of sunlight to form ozone. When the NO_2_ level is high, it can further react with ozone and clean it. Whereas, in the cleaner areas with low NO_2_ levels, the ozone concentration becomes high as there is no NO_2_ to further exploit it.^34^ Controlling the VOC emissions from vehicles and from the combustion of solid fuels is crucial to control ozone pollution. An amendment in the transport system with cleaner fuels and battery in vehicles and controlling emissions due to industries, constructions, and waste burning is crucial to alleviate the air quality.

## CONCLUSIONS

In this work, experiments using various machine learning techniques have been performed to monitor the impact of various air pollutants during lockdown amid COVID-19 in Delhi, and which pollutant types contribute to the deaths. Further, a comparison between the concentration of various air pollutants and AQI during the lockdown period and the last 2 years has been presented. From this study, it has been inferred that the concentration of all the pollutants has decreased significantly apart from ozone and toluene, with an increase of 94.42% and 95.98%, respectively. The highest percentages of decrease of 63.68%, 56.56%, and 93.16% exist for the concentration of NO_2_, SO_2_, and benzene, respectively. Machine learning techniques have identified ozone, NH_3_, NO_2_, and PM_10_ as indicators that may impact deaths caused by COVID-19. The parameter that holds importance here is ozone, as an increase in its concentration has been noted, and further it has been observed that it impacts the COVID-19 mortality rate. In the future, preliminary precautions can be taken to mitigate the levels of VOC, which further impact the ozone concentration.
